# Observations on the Umbilico-Mesenteric Vessels

**Published:** 1803-02-01

**Authors:** 

**Affiliations:** Professor in the Medical School of Paris


### *Observations on the Umbilico-Mesenteric Vessels, by* Cit. Chaussier, *Professor in the Medical School of Paris; read at the Medical School.*

The fœtus, he observes, is connected to the placenta by the umbilical cord, consisting of two arteries and a large vein, also by a mucous substance, and covered exteriorly by a membranous prolongation; we generally find also the uracus, a small membranous canal proceeding from the superior part of the bladder accompanying the umbilical vessels, and running along the cord opening into a membranous neck called alantois. The disposition and uses of these vessels are too well known to require any particular description; but when we cautiously open the abdomen of certain animals who have died in utero, or or shortly after birth, we may perceive two other small thread-like vessels, which are sent off from the mesentery, and running toward the umbilicus, where they seem to terminate, and for which reason they have been termed umbilico-mesenteric vessels. On injecting these vessels with oil of turpentine or mercury, we discover that one of them is an artery sent off by the superior mesenteric, the other somewhat larger, a vein going to the right and opening into the mesenteric, near the trunk of the vena portæ; we may observe too, that these vessels do not simply run from the mesentery to the navel, as their name would seem to indicate, but after proceeding the entire length of the umbilical cord, and arriving at the concave surface of the placenta, they separate, divide, and ramify, on a membranous bladder of an oval shape, lying between the chorion and amnios, and perfectly distinct from the alantois. This vesicula, which had been remarked by Needham, and which had got the name of vesicula umbilicalis, is very remarkable in the first periods of gestation, when it is found filled with a diaphonous fluid; the quantity of this fluid diminishes as gestation advances, and after a certain time the sac, or bladder, becomes completely empty, the sides collapse, and nothing is perceivable but a membrane with ramifications of vessels running on its surface. After birth, the portion of these vessels which lie within the abdomen become obliterated, so as to leave no trace whatever of their existence. This appearance, as we have now described it, had been in part seen by Fabricius, Severenus, Bartholin, Duverney, &c. in the dog, cat, lion; and Needham, who described it with much accuracy, found it in the rabbit. Cit. Chausier has seen it in birds, &c. but it is desirable to know if it exists in the human subject. If we confined our researches to human fœtuses arrived at their full term, we should be led to declare that they did not exist; they have been, however, found by many anatomists; and Haller found them in a fœtus arrived at the full term.

Cit. Chaussier relates, in the Memoirs of the Academy of Dijon for 1782, that he found in a fœtus of seven or eight months, not only the artery but the vein; and in last Germinal he shewed to the Society of the Medical School these vessels, which he had injected, and traced to the portion of cord without the abdomen, in a fœtus which died some hours after birth. It is, no doubt, difficult to find these vessels at a period so much advanced; it would seem as if this apparatus existed from the very earliest periods riods of developement of the fœtus; Cit. Chaussier always found it in the fœtuses he had occasion to examine of 30, 40, or 60 days; in the smallest embryos this bladder equals in size a small cherry. Albinus, Sandifort, but especially Wresburg and Hunter, have described these appearances; and their observations have left no ground for doubt upon the subject.

From these and many analogous considerations, which Cit. Chaussier has frequently explained in his public Lectures, he thinks that the vesicula umbilicalis, as well as its vessels, exist equally in all animals; that this apparatus is of *some* use in the nutrition and developement of the embryo; but becoming useless in the later periods, it is obliterated and effaced, as we find to be the case in other temporal parts, as the membrana pupillaris, &c. Any traces of them existing at a later period is merely accidental.

*Paris, Jan.* 2, 1803.

